# Cardiopulmonary exercise testing and pulmonary function testing for predicting the severity of CTEPH

**DOI:** 10.1186/s12890-021-01668-3

**Published:** 2021-10-18

**Authors:** Hanqing Zhu, Xingxing Sun, Yuan Cao, Bigyan Pudasaini, Wenlan Yang, Jinming Liu, Jian Guo

**Affiliations:** 1 Department of Pulmonary Function Test, Shanghai Pulmonary Hospital, School of Medicine, Tongji University, Shanghai, 200092 China; 2 Department of Internal Medicine, Columbia Bainuo Clinic, Shanghai, 200040 China

**Keywords:** CPET, PFT, CTEPH

## Abstract

**Background:**

Cardiopulmonary exercise testing (CPET) and pulmonary function testing (PFT) are noninvasive methods to evaluate the respiratory and circulatory systems. This research aims to evaluate and monitor chronic thromboembolic pulmonary hypertension (CTEPH) noninvasively and effectively by these two methods. Moreover, the research assesses the predictive value of CPET and PFT parameters for severe CTEPH.

**Methods:**

We used data from 86 patients with CTEPH (55 for test set, and 31 for validation set) at the Shanghai Pulmonary Hospital Affiliated to Tongji University. The clinical, PFT and CPET data of CTEPH patients of different severity classified according to pulmonary artery pressure (PAP) (mm Hg) were collected and compared. Logistic regression analysis was performed to appraise the predictive value of each PFT and CPET parameter for severe CTEPH. The performance of CPET parameters for predicting severe CTEPH was determined by receiver operating characteristic (ROC) curves and calibration curves.

**Results:**

Data showed that minute ventilation at anaerobic threshold (VE @ AT) (L/min) and oxygen uptake at peak (VO_2_ @ peak) (mL/kg/min) were independent predictors for severe CTEPH classified according to PAP (mm Hg). Additionally, the efficacy of VE @ AT (L/min) and VO_2_ @ peak (mL/kg/min) in identifying severe CTEPH was found to be moderate with the area under ROC curve (AUC) of 0.769 and 0.740, respectively. Furthermore, the combination of VE @ AT (L/min) and VO_2_ @ peak (mL/kg/min) had a moderate utility value in identifying severe CTEPH with the AUC of 0.843.

**Conclusion:**

Our research suggests that CPET and PFT can noninvasively and effectively evaluate, monitor and predict the severity of CTEPH.

**Supplementary Information:**

The online version contains supplementary material available at 10.1186/s12890-021-01668-3.

## Background

Cardiopulmonary exercise testing (CPET) provides a unique and comprehensive evaluation of respiratory and circulatory systems by detecting the gas exchange and exercise load during exercise [1]. It is considered to be a gold standard of noninvasive measure of cardiorespiratory fitness and exercise capacity [2]. CPET has been widely carried out in patients with pulmonary hypertension (PH), heart failure (HF), chronic obstructive lung disease (COPD), asthma, etc. [3–5]. It has been reported to be of significance in disease diagnosis, therapeutic efficacy evaluation and prognostic assessment. However, the variety of parameters makes it difficult for clinicians to interpret CPET reports accurately.

The pathological characteristics of CTEPH are organized thrombus and vascular remodeling, which can lead to right ventricular failure [6]. Pulmonary endarterectomy (PEA), balloon pulmonary angioplasty (BPA) and PH-targeted medicine are the main therapies of CTEPH [6]. The diagnosis of CTEPH can be achieved by right heart catheterization (RHC), ventilation/perfusion scan (V/Q) and CT pulmonary angiography (CTPA) [7, 8].

Since CTEPH is a kind of progressive disease, it is imperative to evaluate the severity of the CTEPH patients appropriately for timely intervention. It has already been reported that CPET may be used to estimate the severity of PH [9]. Abnormalities noted during CPET were consistent, characteristic and correlated well with primary pulmonary hypertension (PPH) patients’ NYHA class [10, 11]. Stepping on these, we set this study to examine the CPET performance difference between patients with mild-moderate and severe CTEPH.

## Methods

### Ethical approval

The study protocol was reviewed and approved by the Ethics Committee of Shanghai Pulmonary Hospital. Written informed consent was obtained from each patient for inclusion into the study prior to the performance of any study-related procedures.

All methods including CPET, PFT, RHC and blood test were carried out in accordance with relevant guidelines and regulations.

### Patients

This study retrospectively enrolled 86 inpatients with CTEPH who were referred to Shanghai Pulmonary Hospital from November 2015 to December 2019. All patients were definitely diagnosed by RHC. Patients with mean pulmonary artery pressure (mPAP) ≥ 25 (mm Hg) and pulmonary arterial wedge pressure (PAWP) ≤ 15 (mm Hg) were considered to be diagnosed with CTEPH. They also should had thromboembolic disease performance which can be detected by ventilation/perfusion scan or pulmonary angiogram. Patients were excluded from study if they had any evidence of the following: right-to-left cardiac shunt, coexisting lung diseases (identified clinically or on CT scan), FEV1/FVC% < 65%, history of treatment with BPA and PEA. Enrolled patients’ data including demographics, medication, NT-pro BNP, hemodynamics, PFT and CPET were collected. Ethical approval by the medical ethics committee of Shanghai Pulmonary Hospital was obtained.

### CPET

CPET was performed on an electromagnetically braked cycle ergometer (Master Screen CPX, Jaeger crop, Hoechberg, Germany) to record gas exchange data over 10-s intervals by using a breath-by-breath system. The protocol was consisted of the rest phase of 3 min, the unloaded phase of 3 min, the incremental phase, and the recovery phase of 5 min. The patients were instructed to pedal at 55–60 revolutions/min in the unloaded and the incremental phase, and once they reached their limit, entered the recovery phase. Patients could quit at any time if they developed fatigue, dyspnea, chest tightness or any other discomfort during the process. There were three ramp increments models that we used in the incremental phase: 10 W/min, 15 W/min and 20 W/min. We would choose an appropriate model according to the patient's clinical condition and PFT result. Each subject’s exercise time includes the unloaded phase of 3 min and the incremental phase. Some basic information in our research is as follows. In “Mild” group, 8 subjects used 15 W/min ramp increments model, and 2 subjects used 20 W/min ramp increments model. In “Moderate” group, all 10 subjects used 15 W/min ramp increments model. In “Severe” group, 1 subject used 10 W/min ramp increments model, 29 subjects used 15 W/min ramp increments model and 5 subjects used 20 W/min ramp increments model. Each group’s exercise time is as follows: “Mild” group (8.6 ± 1.4 min), “Moderate” group (7.6 ± 1.2 min) and “Severe” group (6.9 ± 1.3 min).

Measurements including load, minute ventilation (VE), carbon dioxide output (VCO_2_), oxygen uptake (VO_2_), oxygen pulse (VO_2_/HR), end-tidal partial pressure for carbon dioxide (PETCO_2_), end-tidal partial pressure for oxygen (PETO_2_), heart rate (HR), breathing reserve (BR), respiratory exchange ratio (RER) and breathing frequency (BF) were recorded and calculated. Anaerobic threshold (AT) which represents the beginning of anaerobic metabolism was determined by the V-slope method and was independently defined by two experienced investigators who have been engaged in clinical and scientific research on CPET for several years. VE/VCO_2_ slope was obtained by linear regression analysis of the relation between VE and VCO_2_. Oxygen uptake efficiency slope (OUES) was computed by linear square regression from the oxygen uptake on the logarithm of the minute ventilation according to the following equation: VO_2_ = a*lgVE + b. Constant “a” is called the OUES. Oxygen uptake efficiency plateau (OUEP) was at 90 s of the highest consecutive values for VO_2_ (mL/min)/VE (L/min).

### PFT

Spirometry and body plethysmography were performed on each patient using standard equipment (Masterscreen-PFT, Jaeger crop, Hoechberg, Germany; Masterscreen-plethysmography, Jaeger crop, Hoechberg, Germany). Forced vital capacity (FVC), forced expiratory volume in 1 s (FEV1), residual volume (RV), total lung capacity (TLC) and diffusing capacity for carbon monoxide (DLCO) were determined by standard procedures [12, 13]. For each patient, data were presented in absolute terms and normalized to percentage of normal predicted (% Pred). All measurements were calculated using accepted equations for Chinese adults [14].

### Statistical analysis

Data were analyzed by using SPSS 22.0 and GraphPad Prism 6. The data were presented as mean ± SD, median (interquartile range), or n. One-way ANOVA test, Kruskal–Wallis test, Unpaired t test, Mann–Whitney U test, chi-square test, univariate logistic regression analysis and multivariate logistic regression analysis were used according to the corresponding situation. A two-tailed *P* < 0.05 was considered statistically significant.

## Results

### Characteristics of the CTEPH subjects

55 patients with CTEPH were involved in the test set. They were divided into “Mild”, “Moderate” and “Severe” group according to PAP (mm Hg). Mild: 35 > PAP (mm Hg) ≥ 25; Moderate: 45 > PAP (mm Hg) ≥ 35; Severe: PAP (mm Hg) ≥ 45.

The characteristics of all groups were summarized in Table 1. The “Severe” group had the highest value of NT-proBNP (pg/mL) (1082 (642.0, 2674)) when compared with the “Mild” (143.0 (63.8, 286.7)) and “Moderate” (648.5 (266.3, 2049) group. The values of PAP (mm Hg) of the “Mild”, “Moderate” and “Severe” groups were statistically different (28.6 ± 3.3 *vs.* 39.5 ± 3.9 vs. 56.4 ± 8.0 mm Hg; *P* < 0.001). Additionally, the values of PVR (wood u) and RAP (mm Hg) of the three groups were also statistically different. PVR (wood u) values of the “Mild”, “Moderate” and “Severe” group were listed (3.7 ± 1.3 *vs.* 7.6 ± 2.4 vs. 10.9 ± 3.8 wood u; *P* = 0.011). And RAP (mm Hg) values of the “Mild” (0.5 (0, 4.5)), “Moderate” (1.0 (0, 1.3)) and “Severe” (4.0 (2.0, 7.0)) group were also listed.

**Table 1:** Characteristics of CTEPH subjects of different severity

Variables	Total	Mild	Moderate	Severe	*P*
*Clinical characteristics*					
Age (years)	61.2 ± 11.2	65.5 ± 9.9	59.8 ± 9.3	60.4 ± 12.0	0.418
Sex, n (female/male)	31/24	4/6	7/3	20/15	0.396
Height (m)	1.6 ± 0.1	1.7 ± 0.1	1.6 ± 0.1	1.6 ± 0.1	0.527
Weight (kg)	63.3 ± 12.9	65.3 ± 12.0	56.4 ± 12.1	64.7 ± 13.1	0.178
Body mass index (kg/m^2^)	23.6 ± 3.2	23.6 ± 2.9	20.8 ± 3.3	24.4 ± 2.9	0.016
WHO classification II/III/IV, n	15/39/1	6/4/0	3/6/1	6/29/0	0.017
*Blood test*					
NT-proBNP (pg/mL)	809.0 (296.9,2284.0)	143.0 (63.8,286.7)	648.5 (266.3,2049.0)	1082.0 (642.0,2674.0)	0.001
*Right heart catheterization parameters*					
PAP (mm Hg)	48.3 ± 13.2	28.6 ± 3.3	39.5 ± 3.9	56.4 ± 8.0	< 0.001
PAWP (mm Hg)	7.0 (4.0,9.0)	8.5 (5.5,10.3)	4.0 (3.0,9.3)	7.0 (4.0,9.0)	0.250
CO (L/min)	5.0 ± 1.4	5.8 ± 1.7	4.8 ± 1.4	4.9 ± 1.3	0.221
CI (L/min/m^2^)	3.0 ± 0.8	3.3 ± 0.8	3.0 ± 0.7	2.9 ± 0.7	0.283
PVR (wood u)	9.0 ± 4.2	3.7 ± 1.3	7.6 ± 2.4	10.9 ± 3.8	0.011
RAP (mm Hg)	3.0 (1.0,6.0)	0.5 (0,4.5)	1.0 (0,1.3)	4.0 (2.0,7.0)	0.001
*Pulmonary function testing parameters*					
FVC (L)	2.7 ± 0.9	2.8 ± 0.8	2.8 ± 1.1	2.6 ± 0.8	0.790
FVC (% Pred)	84.4 ± 15.8	88. 8 ± 15.6	86.3 ± 24.9	82.6 ± 12.5	0.508
FEV1 (L)	2.1 ± 0.7	2.2 ± 0.5	2.2 ± 0.8	2.0 ± 0.7	0.488
FEV1 (% Pred)	79.5 ± 17.1	85.7 ± 14.0	87.0 ± 23.9	75.6 ± 14.7	0.076
FEV1/FVC (%)	75.9 ± 8.8	78.8 ± 9.3	81.7 ± 8.0	73.4 ± 8.0	0.041
RV (L)	2.4 ± 0.7	2.5 ± 0.9	2.6 ± 0.3	2.3 ± 0.7	0.562
RV (% Pred)	126.8 ± 34.3	122.6 ± 46.1	134.9 ± 27.4	125.7 ± 32.9	0.700
TLC (L)	5.1 ± 1.3	5.3 ± 1.5	5.4 ± 1.2	5.0 ± 1.3	0.629
TLC (% Pred)	100.6 ± 19.0	101.7 ± 26.0	104.7 ± 15.4	99.1 ± 18.0	0.698
RV/TLC (%)	47.3 (41.8,53.8)	48.3 (39.0,53.8)	49.0 (39.4,61.0)	47.2 (42.0,56.1)	0.910
SB DLCO (% Pred)	81.8 ± 19.2	81.4 ± 25.2	85.0 ± 28.7	81.0 ± 14.0	0.843
*Specific medications*					
PDE-5 inhibitors (n, %)	38 (69.1%)	9 (90.0%)	5 (50.0%)	24 (68.6%)	0.153
ERAs (n, %)	33 (60.0%)	0	7 (70.0%)	26 (74.3%)	0.000
Prostacyclin analogs (n, %)	1 (1.8%)	1 (10.0%)	0	0	0.101
sGC activators	3 (5.5%)	0	0	3 (8.6%)	0.404
Combination (n, %)	20 (36.4%)	0	2 (20.0%)	18 (51.4%)	0.006

Range for “Mild”: 35 > PAP (mm Hg) ≥ 25; range for “Moderate”: 45 > PAP (mm Hg) ≥ 35; range for “Severe”: PAP (mm Hg) ≥ 45. The data are presented as mean ± SD, median (interquartile range), or n. Statistical analysis of characteristics of “Mild”, “Moderate” and “Severe” was analyzed with One-way ANOVA test, Kruskal–Wallis test or chi-square test, and was presented as “*P*”. WHO = World Health Organization; BNP = brain natriuretic peptide; PAP = pulmonary artery pressure; PAWP = pulmonary arterial wedge pressure; CO = cardiac output; CI = cardiac index; PVR = pulmonary vascular resistance; RAP = right atrial pressure; FVC = forced vital capacity; FEV1 = forced expiratory volume in 1 s; RV = residual volume; TLC = total lung capacity; SB DLCO = carbon monoxide diffusing capacity; PDE-5 inhibitors = phosphodiesterase type 5 inhibitors; ERAs = endothelial receptor antagonists; sGC activators = soluble guanylate cyclase activators

Phosphodiesterase type 5 (PDE-5) inhibitors, endothelial receptor antagonists (ERAs), prostacyclin analogs and soluble guanylate cyclase (sGC) activators were therapeutic agents used by the CTEPH patients, which included sildenafil, tadalafil, vadenafil, ambrisentan, bosentan, beraprost and iloprost. Details were listed in Table 1.

### CPET and PFT performance differences in subjects with CTEPH

FEV1/FVC (%) was the only parameter that was statistically different between the “Severe”, “Mild” and “Moderate” group (78.8 ± 9.3 *vs.* 81.7 ± 8.0 *vs.* 73.4 ± 8.0%; *P* = 0.041). Details were listed in Table 1.

16 parameters were found to be statistically different among the “Mild”, “Moderate” and “Severe” group. They were listed as followed: Load @ Peak (W), VO_2_ @ Rest (mL/kg/min), VO_2_ @ Peak (mL/kg/min), VE @ AT (L/min), BR @ Rest (%), BR @ AT (%), VE/VCO_2_ @ AT, VE/VCO_2_ @ Peak, VE/VO_2_ @ AT, PETCO_2_ @ Rest (mm Hg), PETCO_2_ @ AT (mm Hg), PETCO_2_ @ Peak (mm Hg), PETO_2_ @ AT (mm Hg), PETO_2_ @ Peak (mm Hg), VE/VCO_2_ slope and LOWEST VE/VCO_2_. Details were listed in Tables 2 and 3.

**Table 2:** Comparison of the CPET parameters of CTEPH subjects of different severity

Variables	Rest				AT				Peak			
	Mild	Moderate	Severe	*P*	Mild	Moderate	Severe	*P*	Mild	Moderate	Severe	*P*
Load (W)					40.0 (35.5,48.5)	36.0 (28.0,41.0)	32.0 (24.0,42.0)	0.177	74.3 (66.4,106.0)	65.7 (56.0,84.7)	55.0 (42.7,72.0)	0.009
HR	84.1 ± 14.3	81.3 ± 11.5	82.4 ± 12.5	0.881	109.7 ± 22.7	110.0 ± 13.0	113.7 ± 16.1	0.727	135.0 ± 19.1	132.2 ± 20.2	128.6 ± 16.4	0.565
VO_2_ (mL/kg/min)	4.0 ± 0.5	5.1 ± 0.9	4.4 ± 0.8	0.017	9.3 ± 2.8	9.9 ± 1.2	9.1 ± 1.6	0.518	13.6 ± 3.5	12.8 ± 1.6	10.8 ± 2.2	0.013
VO_2_ (% pred)	17.1 ± 2.5	20.8 ± 4.4	18.8 ± 5.3	0.229	39.1 ± 8.3	41.1 ± 9.6	38.9 ± 12.5	0.872	57.4 ± 10.8	53.1 ± 12.1	46.4 ± 15.5	0.077
VO_2_/HR (mL)	3.2 ± 0.8	3.5 ± 0.8	3.5 ± 0.8	0.525	5.5 ± 1.6	5.0 ± 0.9	5.2 ± 1.3	0.647	6.7 ± 2.1	5.5 ± 1.1	5.5 ± 1.4	0.083
VE (L/min)	12.3 ± 2.5	13.1 ± 2.8	14.3 ± 3.2	0.158	26.0 ± 8.0	26.2 ± 4.5	32.2 ± 6.5	0.004	46.5 ± 12.4	43.2 ± 12.0	45.2 ± 12.6	0.835
BF (/min)	20.8 (16.9,24.7)	18.1 (15.3,23.3)	20.1 (17.3,23.0)	0.350	26.0 (18.8,34.5)	20.0 (17.8,31.3)	27.0 (23.0,31.0)	0.178	32.0 (25.5,45.1)	29.3 (23.9,37.9)	31.7 (29.3,35.7)	0.580
BR (%)	85.8 (84.0,88.3)	85.1 (83.0,87.8)	80.0 (77.8,85.3)	0.029	68.7 (65.9,76.7)	70.0 (58.1,77.0)	54.1 (42.2,67.3)	0.011	47.9 (40.4,56.7)	50.6 (28.8,58.0)	35.2 (25.4,54.1)	0.348
RER	0.88 ± 0.07	0.86 ± 0.06	0.85 ± 0.07	0.449	0.95 ± 0.07	0.95 ± 0.08	0.96 ± 0.06	0.815	1.15 ± 0.10	1.10 ± 0.10	1.10 ± 0.08	0.061
VE/VCO_2_	55.3 ± 9.9	54.7 ± 5.0	60.53 ± 9.1	0.085	46.6 ± 10.9	51.3 ± 8.2	58.9 ± 12.3	0.013	47.0 ± 11.1	55.7 ± 13.4	62.5 ± 15.2	0.010
VE/VO_2_	47.8 ± 5.9	47.3 ± 5.3	51.0 ± 6.6	0.157	44.3 ± 10.2	48.9 ± 10.7	57.5 ± 15.4	0.010	53.5 ± 12.2	61.9 ± 19.0	67.6 ± 19.7	0.106
PETCO_2_ (mm Hg)	27.7 ± 4.7	25.8 ± 2.1	23.9 ± 3.2	0.037	29.3 ± 5.4	25.2 ± 3.8	22.8 ± 4.4	0.004	28.5 ± 5.8	23.2 ± 5.1	21.3 ± 5.2	0.007
PETO_2_ (mm Hg)	119.8 ± 3.2	122.3 ± 3.4	122.9 ± 3.9	0.074	119.9 ± 5.6	125.1 ± 4.7	126.9 ± 4.9	0.006	124.7 ± 4.3	130.2 ± 5.6	130.2 ± 5.7	0.020

Range for “Mild”: 35 > PAP (mm Hg) ≥ 25; range for “Moderate”: 45 > PAP (mm Hg) ≥ 35; range for “Severe”: PAP (mm Hg) ≥ 45. The data are presented as mean ± SD or median (interquartile range). Statistical analysis of characteristics of “Mild”, “Moderate” and “Severe” was analyzed with One-way ANOVA test or Kruskal–Wallis test, and was presented as “*P*”. VO_2_ = oxygen uptake; HR = heart rate; VE = minute ventilation; BF = breathing frequency; BR = breathing reserve; RER = respiratory exchange ratio; VCO_2_ = carbon dioxide output; PETCO_2_ = end-tidal partial pressure for carbon dioxide; PETO_2_ = end-tidal partial pressure for oxygen

**Table 3:** Comparison of the CPET parameters of CTEPH subjects of different severity

Variables	Mild	Moderate	Severe	*P*
OUES (L/min/log(L/min))	1.3 ± 0.4	1.0 ± 0.3	1.0 ± 0.4	0.066
OUEP (mL/L)	23.4 (21.0, 27.9)	22.7 (21.4, 23.8)	21.3 (19.1, 22.7)	0.077
VE/VCO_2_ slope	40.9 (32.3, 53.0)	48.4 (41.1, 68.7)	61.7 (49.9, 78.5)	0.005
LOWEST VE/VCO_2_	43.7 ± 9.1	48.6 ± 5.8	55.0 ± 10.1	0.008

Range for “Mild”: 35 > PAP (mm Hg) ≥ 25; range for “Moderate”: 45 > PAP (mm Hg) ≥ 35; range for “Severe”: PAP (mm Hg) ≥ 45. The data are presented as mean ± SD or median (interquartile range). Statistical analysis of characteristics of “Mild”, “Moderate” and “Severe” was analyzed with One-way ANOVA test or Kruskal–Wallis test, and was presented as “*P*”. OUES = oxygen uptake efficiency slope; OUEP = oxygen uptake efficiency plateau; VE = ventilation; VCO_2_ = carbon dioxide output

### Predictive value of the CPET and PFT parameters for severe CTEPH

**Fig. 1:**
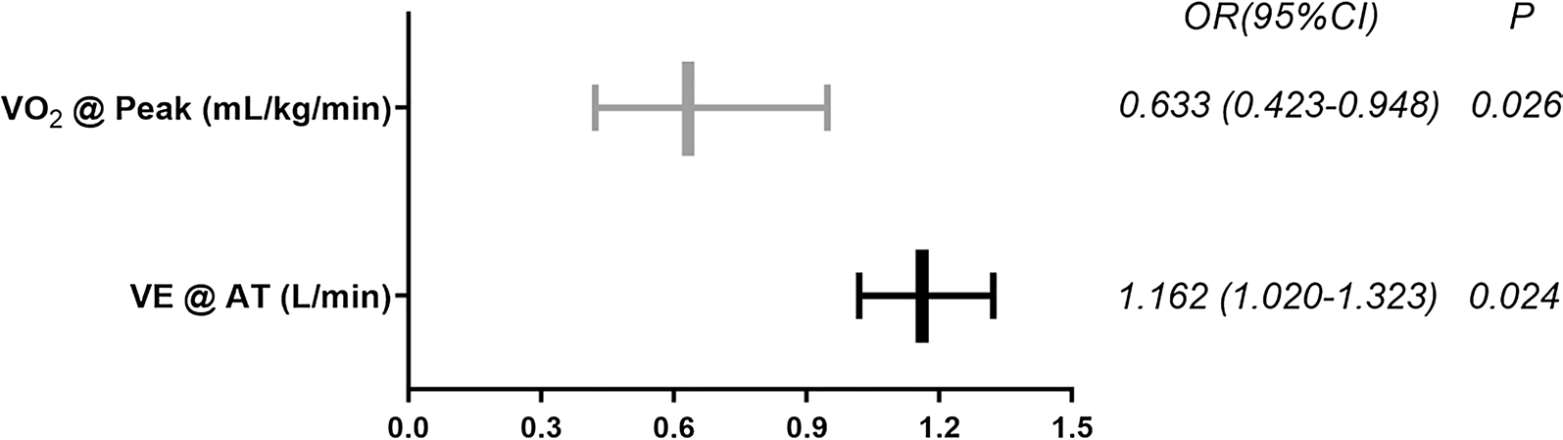
Predictors of severe CTEPH on multivariable analysis of CPET and PFT parameters. Range for “Severe”: PAP (mm Hg) ≥ 45. Results are expressed as odds ratio (OR) with 95% confidence interval (95% CI). VE = minute ventilation; VO_2_ = oxygen uptake

55 patients with CTEPH in the test set were re-grouped into “Mild-Moderate” and “Severe” group to analyze predictors for severe CTEPH. All the CPET and PFT parameters indicated were analyzed with the univariate analysis for the severe CTEPH, and 20 parameters were found to have a *P* < 0.05. They were listed in Additional file 1: Table S1. Considering the sample size, 4 parameters with the minimum *P* value were fitted into the multivariate analysis, including VE @ AT (L/min) (OR 1.169, *P* = 0.004), PETCO_2_ @ AT (mm Hg) (OR 0.809, *P* = 0.005), VO_2_ @ peak (mL/kg/min) (OR 0.627, *P* = 0.005) and LOWEST VE/VCO_2_ (OR 1.129, *P* = 0.006). By using multivariate logistic regression analysis, it was found that VE @ AT (L/min) (OR 1.162, *P* = 0.024) and VO_2_ @ peak (mL/kg/min) (OR 0.633, *P* = 0.026) were independent predictors for the severe CTEPH. Details were listed in Table 4 and Fig. 1.

**Table 4:** Predictors of severe CTEPH on univariable and multivariable analysis of CPET and PFT parameters

Variables	Univariate analysis			Multivariate analysis		
	OR	*P*	95% CI	OR	*P*	95% CI
VE @ AT (L/min)	1.169	0.004	1.051–1.300	1.162	0.024	1.020–1.323
PETCO_2_ @ AT (mm Hg)	0.809	0.005	0.698–0.938	1.023	0.901	0.713–1.469
VO_2_ @ peak (mL/kg/min)	0.627	0.005	0.452–0.870	0.633	0.026	0.423–0.948
LOWEST VE/VCO_2_	1.129	0.006	1.036–1.230	1.048	0.645	0.859–1.279

Range for “Severe”: PAP (mm Hg) ≥ 45. CPET and PFT parameters were all analyzed with univariate logistic regression analysis, and 4 parameters with minimum *P* value entered in the multivariate logistic regression analysis. Results are expressed as odds ratio (OR) with 95% confidence interval (95% CI). VE = minute ventilation; VO_2_ = oxygen uptake; VCO_2_ = carbon dioxide output; PETCO_2_ = end-tidal partial pressure for carbon dioxide

**Fig. 2:**
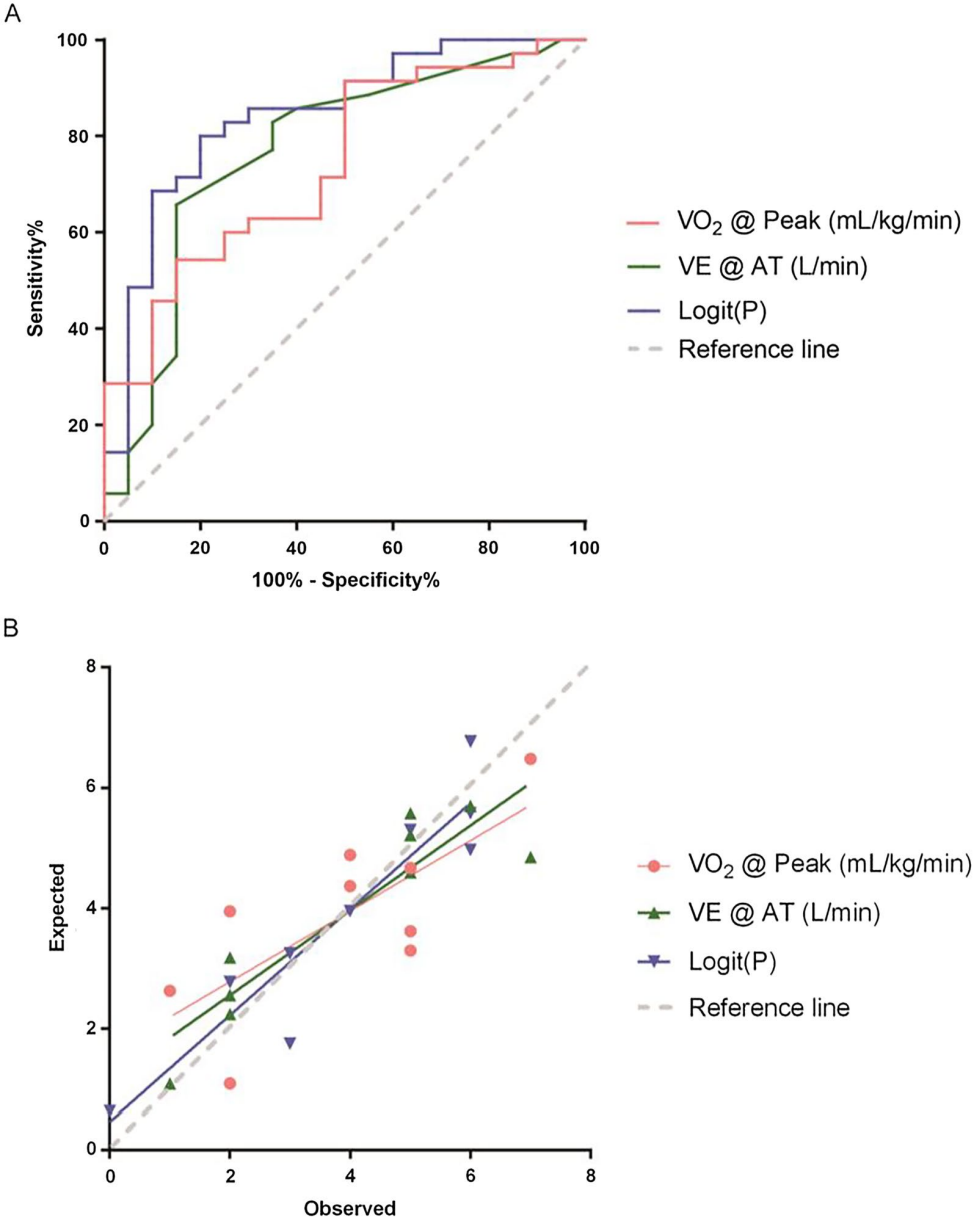
Performance of CPET parameters for the prediction of severe CTEPH. **A** Receiver operating characteristic (ROC) curves for single factor and multiple factors that predict severe CTEPH. **B** Calibration curves for single factor and multiple factors that predict severe CTEPH

Multivariate logistic regression was used to establish a prediction model for predicting severe CTEPH: Logit(*P*) = Log(P/1 − P) = 1.753 + 0.168 * VE @ AT (L/min) − 0.505 * VO_2_ @ peak (mL/kg/min). To evaluate the ability of the VE @ AT (L/min), VO_2_ @ peak (mL/kg/min) and the prediction equation to discriminate severe CTEPH, ROC curves and calibration curves analysis were performed. Details were listed in Table 5 and Fig. 2. It should be noted that the AUC of the prediction equation was better than that for each parameter, indicating that the equation based on two parameters could improve the prediction performance for severe CTEPH.

**Table 5:** Predictive value of single factor and multiple factors for severe CTEPH

Variables	AUC	95% CI	*P*	Cutoff-point value	Sensitivity	Specificity	Youden index
VE @ AT (L/min)	0.769	0.630–0.908	0.001	29.5	65.7%	85.0%	0.507
VO_2_ @ Peak (mL/kg/min)	0.740	0.606–0.874	0.003	13.0	50.0%	91.4%	0.414
Logit(*P*)	0.843	0.732–0.954	0.000	0.716	68.6%	90.0%	0.586

Range for “Severe”: PAP (mm Hg) ≥ 45. AUC = area under ROC curve; CI = confidence interval; VE = minute ventilation; VO_2_ = oxygen uptake

Additionally, the optimum cut-off value of Logit(*P*) ≥ 0.716 to predict severe CTEPH was determined by using ROC analysis (AUC = 0.843, 95% CI = 0.732 to 0.954, Youden index = 0.586). We calculated the value of Logit(*P*) of each subject in the validation datasets to validate the results. Only 4 of 31 patients with CTEPH of the validation set were ambiguous, and the accuracy was 87.10%. Details were listed in Table 6.

**Table 6:** Characteristics of subjects with Logit(*P*) < 0.716 and Logit(*P*) > 0.716 calculated by the predication equation in validation set

Variables	Logit(*P*) < 0.716	Logit(*P*) > 0.716	*P*
Age (years)	54.0 ± 13.0	60.8 ± 6.7	0.102
Sex, n (female/male)	14/5	7/5	0.075
Height (m)	1.6 ± 0.1	1.6 ± 0.1	0.348
Weight (kg)	61.2 ± 10.2	66.4 ± 10.9	0.187
Body mass index (kg/m^2^)	23.7 ± 3.8	24.5 ± 2.6	0.501
WHO classification II/III/IV, n	10/9/0	2/9/1	0.082
NT-proBNP (pg/mL)	203.0 (79.0,634.0)	1305.0 (659.0,2284.0)	0.002
PAP (mm Hg)	36.3 ± 11.7	53.0 ± 4.6	< 0.0001
PAWP (mm Hg)	7.5 ± 3.7	6.3 ± 2.6	0.338
CO (L/min)	4.9 ± 1.0	4.2 ± 1.0	0.075
CI (L/min/m^2^)	3.0 ± 0.5	2.5 ± 0.5	0.017
PVR (wood u)	6.3 ± 2.6	11.5 ± 3.6	< 0.0001
RAP (mm Hg)	3.0 (2.0,5.0)	7.5 (3.5,8.8)	0.045
VE @ AT (L/min)	26.0 ± 6.4	35.6 ± 7.1	0.001
VO_2_ @ Peak (mL/kg/min)	13.8 ± 2.7	11.1 ± 1.8	0.005

The data are presented as mean ± SD, median (interquartile range), or n. Statistical analysis of characteristics of “Logit(*P*) < 0.716” and “Logit(*P*) > 0.716” was analyzed with Unpaired t test, Mann–Whitney U test or chi-square test, and was presented as “*P*”. WHO = World Health Organization; BNP = brain natriuretic peptide; PAP = pulmonary artery pressure; PAWP = pulmonary arterial wedge pressure; CO = cardiac output; CI = cardiac index; PVR = pulmonary vascular resistance; RAP = right atrial pressure; VE = minute ventilation; VO_2_ = oxygen uptake

## Discussion

We retrospectively analyzed the clinical, hematological, PFT and CPET data of 86 patients with CTEPH. Part of the patients were randomly classified into the validation set (a total of 31), and the remaining 55 patients were classified into the test set. Patients with CTEPH in the test set were divided into “Mild”, “Moderate” and “Severe” group according to PAP (mm Hg) detected by RHC to find the parameters that can predict severe CTEPH [15, 16]. Results exhibited that there were statistical differences in the CPET performance of patients with different severity of CTEPH. For example, the parameters related with the subjects’ exercise capacity were statistically different: Load @ Peak (W), VO_2_ @ Rest (mL/kg/min) and VO_2_ @ Peak (mL/kg/min). Several parameters associated with subjects’ ventilatory and gas exchange efficiency were also statistically different, including VE @ AT (L/min), BR @ Rest (%), BR @ AT (%), VE/VCO_2_ @ AT, VE/VCO_2_ @ Peak, VE/VO_2_ @ AT, PETCO_2_ @ Rest (mm Hg), PETCO_2_ @ AT (mm Hg), PETCO_2_ @ Peak (mm Hg), PETO_2_ @ AT (mm Hg), PETO_2_ @ Peak (mm Hg), VE/VCO_2_ slope and LOWEST VE/VCO_2_.

The patients with CTEPH in the test set were regrouped into “Mild-Moderate” and “Severe” group to analyze predictors for severe CTEPH by univariate and multivariate analysis. The results indicated that VE @ AT (L/min) and VO_2_ @ Peak (mL/kg/min) were independent predictors for severe CTEPH. Combining these two parameters, we got a prediction equation for severe CTEPH. ROC curves and calibration curves proved that the prediction equation was good in discrimination and calibration. Additionally, the prediction equation’s application in the validation set further confirmed its efficiency.

CTEPH which is kind of pulmonary vascular disease is associated with hypoperfusion of the ventilated alveoli, and it leads to the alveoli with non-occluded capillaries must be ventilated to a proportionately greater degree than normal to remove CO_2_ and to maintain PaCO_2_, PaO_2_ at appropriate levels [17]. Due to increased physiological dead space and low-PaO_2_ driven ventilation, increased ventilation was observed in patients with CTEPH. The increase in VE was observed in patients with CTEPH at rest phase and to a greater degree during exercise phase. Compared with pulmonary arterial hypertension (PAH) patients, CTEPH patients have higher VE values and lower BR (%) values at AT phase [18]. In our study, it was also observed that VE values at AT phase were closely related with the severity of CTEPH. During exercise, AT is termed as the level of VO_2_ above which aerobic energy production is supplemented by anaerobic mechanisms, lactate continuously increases and metabolic acidosis occurs. Above the AT, VE increases disproportionately to the metabolic requirement to emit CO_2_ to alleviate metabolic acidosis. This may explain to a certain extent, the VE @ AT can more accurately reflect the pathological ventilation of patients with CTEPH than VE @ Peak [19].

CPET can be used for the diagnosis/differential diagnosis, prognostic evaluation and treatment evaluation of CTEPH. Compared with healthy subjects, patients with CTEPH had higher values of VE/VO_2_ @ AT, VE/VCO_2_ @ AT, P(c-ET)CO_2_ while had lower values of PETCO_2_ @ AT. Among these parameters, P(c-ET)CO_2_ was a diagnostic parameter of CTEPH with the highest sensitivity (85.7%) and specificity (88.2%) [20]. Ventilatory efficiency parameters including P(c-ET)CO_2_, VD/VT @ Peak, VE/VCO_2_ slope, VE/VCO_2_ @ AT, OUEP and OUE @ AT can help to distinguish CTEPH from idiopathic pulmonary arterial hypertension (IPAH) [18, 21–23]. The lowest VE/VCO2 ratio could be used to predict CTEPH in patients with chronic PE [24]. Godinas et al. reported that in distal CTEPH patients, higher values of VD/VT were associated with worse survival [25]. Jin et al. reported that after BPA, patients with inoperable CTEPH had better CPET and PFT performance, including improvements in Load @ Peak, VO_2_ @ Peak, OUES, FVC, FEV1 and MVV [26].

For the first time, we have evaluated the CPET performance in CTEPH patients of different severity. For patients who have already been diagnosed with mild CTEPH by the RHC, it’s necessary to continuously monitor the disease progression. However, patients’ clinical signs and symptoms can be nonspecific [27]. Unlike the invasive method RHC, CPET is a non-invasive tool that can help to identify patients with milder abnormalities. We hope that CPET can be used for routine monitoring of CTEPH patients in the future, and then the application of these parameters and this formula can provide value for guiding patients’ the further examination and treatment.

As a similar non-invasive test, echocardiography makes it possible to estimate the systolic PAP based on the measured tricuspid regurgitation velocity (TRV) at rest and on the presence of additional echocardiographic variables that suggest PH. Although echocardiography is undoubtedly the most important non-invasive test for grading the probability of PH, it also has its limitations: only 90% of PH patients have TRV [28]. In symptomatic patients with a clinical suspicion of PH, the diagnosis of PH is missed by echocardiography in 10–30% of cases, even if indirect signs are taken into consideration [29, 30]. Recently, there was a report that CPET could serve as complementary tool in the diagnosis of CTEPH and can detect CTEPH in patients with normal echocardiography [20]. In the future, perhaps the combination of the two methods will bring the greatest benefits to patients.

In general, our study shows that VE @ AT (L/min) and VO_2_ @ Peak (mL/kg/min) were statistically significant independent predictors for severe CTEPH. The prediction equation Logit(*P*) = 1.753 + 0.168 * VE @ AT (L/min) − 0.505 * VO_2_ @ peak (mL/kg/min) was effective and efficient in discriminating patients with severe CTEPH. There are some methodological limitations. Since most patients are undergoing CPET for the first time, we know very little about their cardiopulmonary function and exercise ability. We just chose a ramp increments model that may be the best based on their clinical condition and PFT result. From the perspective of exercise time, a lower ramp increments model may be more preferable. In the future, if CPET is listed as a regular routine monitoring of patients, so as to establish a file for the patient, this trouble may be avoided. Some of other limitations of this study are its patient sample size, non-randomized nature, single-center design and potential selection bias. Since it’s a retrospective study, it is a bit difficult for us to continuously monitor the CTEPH patient’s disease progression and corresponding CPET and PFT performance. However, we will further verify these research conclusions in the following prospective studies on CTEPH patient’s CPET and PFT performance.

## Conclusions

Our research suggests that CPET and PFT can noninvasively and effectively evaluate, monitor and predict the severity of CTEPH. VE @ AT (L/min) and VO_2_ @ Peak (mL/kg/min) were statistically significant independent predictors for severe CTEPH.

## Supplementary Information

**Additional file 1. Supplementary Table 1**. Predictors of severe CTEPH on univariable analysis of CPET and PFT parameters.

## Data Availability

All the related data are presented in the manuscript.

## Abbreviations

CPET: Cardiopulmonary exercise testing
PFT: Pulmonary function testing
CTEPH: Chronic thromboembolic pulmonary hypertension
PH: Pulmonary hypertension
PPH: Primary pulmonary hypertension
IPAH: Idiopathic pulmonary arterial hypertension
HF: Heart failure
COPD: Chronic obstructive lung disease
PE: Pulmonary embolism
PEA: Pulmonary endarterectomy
BPA: Balloon pulmonary angioplasty
RHC: Right heart catheterization
CTPA: CT pulmonary angiography
V/Q: Ventilation/perfusion scan
ROC: Receiver operating characteristic curves
AUC: Area under the ROC curve
OR: Odds ratio
CI: Confidence interval
WHO: World Health Organization
BNP: Brain natriuretic peptide
PAP: Pulmonary artery pressure
PAWP: Pulmonary arterial wedge pressure
CO: Cardiac output
CI: Cardiac index
PVR: Pulmonary vascular resistance
RAP: Right atrial pressure
FVC: Forced vital capacity
FEV1: Forced expiratory volume in 1 s
RV: Residual volume
TLC: Total lung capacity
SB DLCO: Carbon monoxide diffusing capacity
VO_2_: Oxygen uptake
HR: Heart rate
VE: Minute ventilation
BF: Breathing frequency
BR: Breathing reserve
RER: Respiratory exchange ratio
VCO_2_: Carbon dioxide output
VD/VT: Dead space ventilation as a fraction of tidal volume
PETCO_2_: End-tidal partial pressure for carbon dioxide
PETO_2_: End-tidal partial pressure for oxygen
OUES: Oxygen uptake efficiency slope
OUEP: Oxygen uptake efficiency plateau
VE: Ventilation
VCO_2_: Carbon dioxide output
AT: Anaerobic threshold
