# Supplementary material for: Cardiopulmonary exercise testing and pulmonary function testing for predicting the severity of CTEPH
Source: BMC Pulm Med. 2021 Oct 18;21:324. doi: 10.1186/s12890-021-01668-3 (PMC8521985; doi:10.1186/s12890-021-01668-3)
Supplement: Supplementary file 1 — Additional file 1. Supplementary Table 1. Predictors of severe CTEPH on univariable analysis of CPET and PFT parameters. [file 12890_2021_1668_MOESM1_ESM.docx]

Supplementary Table 1. Predictors of severe CTEPH on univariable analysis of CPET and PFT parameters.

| Variables | Univariate analysis | | |
| --- | --- | --- | --- |
|  | OR | *P* | 95% CI |
| VE @ AT (L/min) | 1.169 | 0.004 | 1.051-1.300 |
| PETCO_2_ @ AT (mm Hg) | 0.809 | 0.005 | 0.698-0.938 |
| VO_2_ @ peak (mL/kg/min) | 0.627 | 0.005 | 0.452-0.870 |
| LOWEST VE/VCO_2_ | 1.129 | 0.006 | 1.036-1.230 |
| BR @ AT (%) | 0.936 | 0.007 | 0.892-0.981 |
| VE/VCO_2_ @ AT | 1.100 | 0.008 | 1.025-1.180 |
| PETO_2_ @ AT (mm Hg) | 1.184 | 0.009 | 1.043-1.345 |
| PETCO_2_ @ Peak (mm Hg) | 0.859 | 0.009 | 0.767-0.963 |
| FEV1/FVC (%) | 0.900 | 0.010 | 0.830-0.975 |
| PETCO_2_ @ Rest (mm Hg) | 0.769 | 0.011 | 0.628-0.943 |
| Load @ Peak (W) | 0.961 | 0.011 | 0.931-0.991 |
| VE/VO_2_ @ AT | 1.098 | 0.012 | 1.021-1.181 |
| VE/VCO_2_ @ Peak | 1.068 | 0.014 | 1.013-1.126 |
| BR @ Rest (%) | 0.866 | 0.019 | 0.768-0.976 |
| VCO_2_ @ Peak (L/min) | 0.997 | 0.027 | 0.995-1.000 |
| VE/VCO_2_ slope | 1.038 | 0.031 | 1.003-1.074 |
| FEV1 (% Pred) | 0.958 | 0.035 | 0.921-0.997 |
| VE/VCO_2_ @ Rest | 1.091 | 0.035 | 1.006-1.184 |
| VO_2_@ Peak (% pred) | 0.955 | 0.038 | 0.915-0.997 |
| RER @ Peak | 0.001 | 0.047 | 0.000-0.929 |

Range for “Severe”: PAP (mm Hg)≥45. CPET and PFT parameters were all analyzed with univariate logistic regression analysis. Results are expressed as odds ratio (OR) with 95% confidence interval (95% CI). VE=minute ventilation; PETCO_2_=end-tidal partial pressure for carbon dioxide; VO_2_=oxygen uptake; BR=breathing reserve; VCO_2_=carbon dioxide output; PETO_2_=end-tidal partial pressure for oxygen; RER=respiratory exchange ratio FVC=forced vital capacity; FEV1=forced expiratory volume in 1s.
